# *Lactobacillus rhamnosus* confers protection against enteropathogenic bacteria by enhancing mucosal immunity and epithelial barrier function

**DOI:** 10.3389/fcimb.2026.1769889

**Published:** 2026-02-23

**Authors:** Xuwen Gao, Jiangfei Zhou, Kai Yan, Yueming Guan, Jiayi Xiang, Yimei Liu, Han Yu, Jing Wang, Yuan Li, Yigang Xu

**Affiliations:** 1 College of Animal Science & Technology, Zhongkai University of Agriculture and Engineering, Guangzhou, China; 2 Shenzhen Key Laboratory of Pathogenic Microbes and Biosafety, School of Public Health (Shenzhen), Sun Yat-Sen University, Shenzhen, China; 3 College of Veterinary Medicine, Jilin Agricultural University, Changchun, China; 4 College of Veterinary Medicine, Zhejiang A&F University, Hangzhou, China

**Keywords:** anti-bacterial infection, intestinal homeostasis, intestine epithelial barrier, *Lactobacillus rhamnosus*, mucosal immunity barrier

## Abstract

**Introduction:**

Probiotics such as *Lactobacillus rhamnosus* represent promising alternatives to antibiotics for combating enteric infections, yet their mechanisms of action remain incompletely understood. This study aimed to elucidate the protective mechanisms of *L. rhamnosus* CIQ249 against enteropathogenic bacterial infection, focusing on the intestinal physical barrier and mucosal immune responses.

**Methods:**

The intestinal colonization ability of CIQ249 was assessed using cFDA-SE labeling and flow cytometry. Growth performance and intestinal morphology were evaluated in mice. Antimicrobial activity of CIQ249 cell-free supernatant was tested against various pathogens, and pathogen damage was visualized by scanning electron microscopy. Protective effects against *Salmonella typhimurium* and *Escherichia coli* K99 were examined in a mouse model. Tight junction protein expression was analyzed *in vitro* and *in vivo* using immunofluorescence, qRT-PCR, and Western blot. Immune responses— including cytokine production, dendritic cell (DC) activation, T follicular helper (Tfh) cell differentiation, and IgA secretion—were assessed by ELISA, flow cytometry, and immunohistochemistry. Transcriptomic changes in porcine intestinal epithelial cells (PIEC) were analyzed by RNA-seq.

**Results:**

CIQ249 demonstrated strong intestinal colonization and increased villus height and the villus-to-crypt ratio, contributing to improved growth performance. Its cell-free supernatant selectively inhibited enteropathogens and induced structural damage in *S. typhimurium* and *E. coli* K99. CIQ249 protected mice from lethal pathogen challenge, preserved intestinal architecture by upregulating tight junction proteins ZO-1 and Claudin-1. It also enhanced mucosal and systemic cytokine levels (IFN-γ, IL-2, IL-4, IL-17, and IL-27), activated DCs, promoted differentiation of CXCR5+CD4+ Tfh and IgA-secreting plasma cells in Peyer’s patches, leading to sIgA production. Transcriptome analysis revealed broad modulation of immune- and barrier-related pathways, with validation of key genes (e.g., IL-10, Masp2, Igf2).

**Conclusion:**

CIQ249 enhances mucosal defense against enteropathogenic bacteria through a dual mechanism—strengthening the epithelial barrier and activating a coordinated DC–Tfh–IgA immune axis. These findings provide a multi-level mechanistic basis for its application as a microecological agent against intestinal infections.

## Introduction

The gut constitutes a complex cellular ecosystem intricately linked to systemic immunity. The intestinal mucosal barrier is essential for maintaining intestinal environment homeostasis and serves as the first line of defense against invading pathogenic microorganisms (Yang et al., 2022). This barrier and the gut mucosal immune system, composed of gut-associated lymphoid tissues, lamina propria, and epithelial cells, are critical for host defense (Kazemifard et al., 2022; Wang et al., 2025; Sun et al., 2023). Disruption of the intestinal barrier by pathogens can lead to inflammation, resulting in diseases such as diarrhea and colitis (He et al., 2022; Högenauer et al., 2006; Kittana et al., 2018). While antibiotics are highly effective against intestinal pathogens, the escalating problem of antimicrobial resistance necessitates the urgent development of alternatives, particularly in livestock and poultry production.

Probiotics, defined as live microorganisms that confer health benefits to the host when administrated in adequate amounts, are part of the commensal gut flora (Sanders et al., 2019). Most probiotics can modulate immune responses and exert beneficial functions (Cristofori et al., 2021; Shi et al., 2020). For example, the presence of *Lactobacilli* in the gut microbiome has been associated with positive responses to immunotherapy (Roviello et al., 2022). Over the past decade, beneficial microorganisms capable of modulating the mucosal immune system have emerged as potential alternatives for enhancing resistance against pathogens infections (Gao et al., 2019; Ding et al., 2019; Huang et al., 2021). Common probiotics include *Lactobacillus*, *Bifidobacterium*, *Bacillus subtilis*, and yeasts (Zhu et al., 2020). *Lactobacillus rhamnosus* is a Gram-positive bacterium prevalent in the digestive tracts of animals and humans. It exhibits strong adhesion to intestinal epithelial cells and robust colonization ability, forming a beneficial biological barrier (Gu et al., 2022). Furthermore, *L. rhamnosus* has been reported to inhibit intestinal pathogens, regulate gut homeostasis, and enhance immunity and growth performance in animals (Inturri et al., 2019). Despite these recognized benefits, the precise mechanisms by which *L. rhamnosus* regulates intestinal homeostasis to combat pathogenic bacterial infection remain insufficiently elucidated.

In this study, we employed the *L. rhamnosus* isolate CIQ249 to elucidate its protective mechanisms against enteric bacterial infections. Our investigation focused on two key aspects: its impact on the intestinal physical barrier and its modulation of the immune barrier. We specifically tested the hypothesis that *L. rhamnosus* enhances mucosal defense through a dual mechanism—by strengthening the epithelial barrier and by orchestrating a defined mucosal immune axis involving dendritic cells (DCs), T follicular helper (Tfh) cells, and IgA production. Furthermore, by conducting RNA-seq analysis of the intestinal transcriptome following oral administration of CIQ249, we aimed to achieve a comprehensive, molecular- and cellular-level understanding of its probiotic activities.

## Materials and methods

### Animals, bacterial strains, and cell

SPF grade BALB/c mice (4–6 weeks old) were purchased from Hangzhou Charles River Co., Ltd. (Hangzhou, China). Mice were fed a standard rodent chow diet (provided by Hangzhou Charles River Co., Ltd.) ad libitum. Feed and water were refreshed every two days. All animal procedures were conducted in compliance with the guidelines for the care and use of laboratory animals approved by the Experimental Animal Ethics Committee of Zhejiang Z&F University (Approval number is ZAFUAC202458). *L. rhamnosus* CIQ249, *Lactobacillus reuteri* (*L. reuteri*), *Enterococcus faecium* (*E*. *faecium*), *Escherichia coli* (*E. coli*) K99, *E. coli* O157, *Listeria monocytogenes* (*L*. *monocytogenes*), *Salmonella typhimurium (S. typhimurium)*, *Shigella* spp., *Vibrio mimicus* (*V. mimicus*), *V. parahaemolyticus*, *Yersinia enterocolitica* (*Y*. *enterocolitica*) were maintained in our laboratory. Porcine intestinal epithelial cells (PIEC) kept in our laboratory were also used in this study.

### Analysis of digestive tract colonization ability

We used 5(6)-carboxyfluorescein diacetate succinimidyl ester (cFDA-SE) to evaluate the digestive tract colonization ability of *L. rhamnosus* CIQ249, according to the assay previously described (Xu and Li, 2007). Briefly, *L. rhamnosus* CIQ249 was cultured and adjusted to 5 × 10^8^ CFU/mL for oral administration. A 10 mM cFDA-SE (ThermoFisher, USA) stock solution was diluted 1:800 and mixed with an equal volume of the CIQ249. After incubation at 37°C for 30 min, the cells were washed and resuspended to 10^10^ CFU/mL. Samples were fixed with 0.75% formaldehyde, and the labeling efficiency was determined by flow cytometry. Mice were randomly divided into two groups (n = 20 per group): normal control group (sterile PBS group) and cFDA-SE-labeled CIQ249 group (2 × 10^9^ CFU/mouse). At 1, 7, 15 and 20 days post-oral administration, five mice per group were euthanized to collect jejunum, ileum, and colon segments from each mouse. Individual section was cut longitudinally, and any visible residual food particles or fecal material was removed from the intestine before being examined for the presence of adhering cFDA-SE-labeled CIQ249. This examination was performed by adding 150 μL of PBS to every 1.0 cm of tissue and dislodging microbes from the mucosal surfaces of the tissues. Cell extracts were fixed with formaldehyde (0.75%, vol/vol) prior to flow cytometry analysis with three technical replicates.

### Growth performance in animals

To assess growth-promoting effects, mice were randomly divided into two groups (n = 10 per group). One group received the strain CIQ249 (2 × 10^9^ CFU per mouse) orally, while the control group received sterile PBS, for 28 consecutive days. Daily feed intake and body weight gain were recorded.

### Structural integrity analysis of intestinal mucosa

BALB/c mice were randomly divided into two groups (n = 5 per group): experimental group (oral CIQ249, 2 × 10^9^ CFU/mouse) and control group (sterile PBS). After 28 days oral administration, three mice per group were euthanized to collect duodenum, jejunum, and ileum. Tissues were fixed in 10% neutral formaldehyde, and processed for Hematoxylin and eosin (H&E) staining. Villus height, crypt depth, and the villus-to-crypt ratio were analyzed.

### *In vitro* bacteriostatic effect of CIQ249 cell-free culture supernatant

Strain CIQ249 was activated, inoculated (1%) into 10 mL sterile MRS broth, and incubated at 37°C for 24 h. The culture supernatant was filter-sterilized and adjusted to pH 7.0. Indicator strains (*E. coli* K99, *E. coli* O157, *L. monocytogenes*, *Shigella* spp., *S. typhimurium*, *L. reuteri*, *E. faecium*, *V. parahaemolyticus*, *Y. enterocolitica*, and *V. mimicus*) were grown to OD_600_ ≈ 1.0. Aliquots (200 µL) of each bacterial culture were spread on appropriate agar media, and 6 mm wells were punched. Wells were filled with CIQ249 supernatant, sterile MRS broth (negative control), or penicillin/streptomycin (10000 U/mL, positive control). Plates were incubated at 37°C for 24 h, and the diameter of inhibition zones was measured. All experiments were performed with three independent biological replicates, each consisting of three technical replicates.

### Detection of CIQ249-free culture supernatant-induced pathogen damage

*L. rhamnosus* CIQ249 was grown in 100 mL MRS broth (1% inoculum) at 37°C for 24 h. *S. typhimurium* and *E. coli* K99 were activated and separately added (1%) to the filter-sterilized CIQ249 supernatant (pH 7.0), followed by incubation at 37°C for 24 h. Controls were incubated in sterile MRS broth. Bacterial morphology was examined by scanning electron microscopy. The experiment was performed with three independent biological replicates, each consisting of three technical replicates.

### Protective effect of CIQ249 against intestinal pathogenic bacteria

Mice were randomly divided into eight groups (n = 10 per group): (1) normal control (sterile PBS) group; (2) CIQ249 alone (2 × 10^9^ CFU/mouse); (3) CIQ249 for 7 days, then infection with *S. typhimurium* (2 × 10^7^ CFU/mouse) group; (4) CIQ249 for 7 days, then infection with *E. coli* K99 (2 × 10^7^ CFU/mouse) group; (5) *S. typhimurium* alone (2 × 10^7^ CFU/mouse group); (6) *E. coli* K99 alone (2 × 10^7^ CFU/mouse) group; (7) co-administration of CIQ249 and *S. typhimurium* (1:1, v/v); (8) co-administration of CIQ249 and *E. coli* K99 (1:1, v/v). Mortality was recorded daily for 8 days. Body weight changes and clinical symptoms (scored according to **Supplementary Table S1**) were monitored. Duodenum, jejunum, and ileum were collected for histopathological examination.

### Expression of tight junction proteins regulated by CIQ249

PIEC was used for assessment *in vitro*. Briefly, PIEC was seeded in 24-well plates. CIQ249, *S. typhimurium* and *E. coli* K99 were grown to OD_600_ ≈ 1.0, washed, and resuspended in DMEM at 1 × 10^8^ CFU/mL. Cells were treated as follows: (1) Control (untreated PIEC); (2) PIEC + CIQ249 (1 × 10^7^ CFU/mL); (3) PIEC + *typhimurium* (1 × 10^7^ CFU/mL); (4) PIEC + *E. coli* K99 (1 × 10^7^ CFU/mL); (5) PIEC pre-incubated with CIQ249 (1 × 10^7^ CFU/mL, 2 h, removed) then challenged with *S. typhimurium* (1 × 10^7^ CFU/mL); (6) PIEC pre-incubated with CIQ249 then challenged with *E. coli* K99; (7) PIEC co-cultured with CIQ249 (5×10^6^ CFU/mL) and *S. typhimurium* (5 × 10^6^ CFU/mL); (8) PIEC co-cultured with CIQ249 (5 × 10^6^ CFU/mL) and *E. coli* K99 (5 × 10^6^ CFU/mL). All treatments lasted 6 h at 37°C. For immunofluorescence assay (IFA), post-treatment, cells were washed, fixed with 4% paraformaldehyde, and blocked with 0.3% BSA. The cells were then incubated with rabbit anti-pig ZO-1 (1:100) or Claudin-1 (1:100) primary antibodies (Proteintech, China), followed by FITC-labeled goat anti-rabbit IgG (1:400, Abcam, UK). Tight junction integrity was observed by fluorescence microscopy. For quantitative RT-PCR analysis (qRT-PCR) of *ZO-1* and *Claudin-1* expression, total RNA was extracted for each treatment group, reverse transcribed, and amplified using SYBR Green (Takara, China) on a 7500 real-time PCR system (ABI, USA) with the primers listed in **Supplementary Table S2**. Gene expression fold changes were calculated using the 2^-ΔΔCt^ method, using β-actin as the internal control. For Western blot analysis of ZO-1 and Claudin-1 expression, cells were lysed, and proteins were separated by 12% SDS-PAGE, transferred to PVDF membranes (Bio-Rad, USA), and blocked with 5% skim milk. Membranes were probed with rabbit anti-pig ZO-1 or Claudin-1 (1:2000, Proteintech) and HRP-labeled goat anti-rabbit IgG (1:5000). Signals were detected using a chemiluminescence kit (Applygen, China). All experiments were performed with three independent biological replicates, each consisting of three technical replicates.

Mice were used for assessment *in vivo*. Briefly, mice were divided into five groups:(1) Control (sterile PBS) group; (2) CIQ249 alone (2 × 10^9^ CFU/mouse for 5 days); (3) *S. typhimurium* or *E. coli* K99 alone (2 × 10^7^ CFU/mouse); (4) CIQ249 for 5 days, then infection with *S. typhimurium* or *E. coli* K99. Intestinal samples were collected for qRT-qPCR and Western blot analysis of ZO-1 and Claudin-1, using rabbit anti-mouse ZO-1 of Claudin-1 primary antibodies (Absin, China) and HRP-labeled goat anti-rabbit IgG secondary antibody. All experiments were performed with three independent biological replicates, each consisting of three technical replicates.

### Regulation of cytokines by oral CIQ249 *in vivo*

Mice (n = 15 per group) received oral *L. rhamnosus* CIQ249 (200 μL of 1 × 10^10^ CFU/mL) or sterile PBS (control). At 0, 7, 14, 21 and 28 days post- administration, three mice per group were euthanized to collect serum and intestinal mucus. Levels of IFN-γ, IL-2, IL-4, IL-17, and IL-27, as well as IgA in serum and sIgA in intestinal mucus were measured using commercial ELISA kit (TransGen Biotech, China) with three technical replicates.

### Activation of dendritic cells and differentiation of IgA-secreting cells in Peyer’s patches induced by CIQ249

These experiments were carried out in accordance with previously described protocols (Jia et al., 2020). To detect dendritic cells (DCs) activation in Peyer’s patches (PPs), the PPs were isolated from the intestines of mice in the CIQ249 and PBS control groups on day 7 post oral administration. The tissues were grinded homogenized in 5 mL of pre-cooled Hank’s Balanced Salt Solution (HBSS). The resulting cell suspension was filtered through a 300-mesh stainless steel sieve and centrifuged at 500 × g for 10 min. After centrifugation, the cell pellet was resuspended in 8 mL HBSS, carefully layered onto 5 mL of 70% Per-coll (Sigma, USA) solution, and centrifuged at 500 × g for 20 min. Cells on the interface were collected, resuspended in HBSS at a concentration of 10^6^/mL, and incubated with anti-CD11c antibody (Abcam, USA). After washing, the cells were stained with FITC-conjugated anti-CD40 or anti-CD86 antibodies (Abcam, USA), and analyzed by flow cytometry. The expression of Bcl-6 in T lymphocytes within the PPs was detected by immunohistochemistry (IHC). Briefly, PPs were isolated from mice in the CIQ249 and PBS control groups, fixed in 10% formaldehyde, and embedded in paraffin. The tissue sections were then prepared and incubated with a mouse anti-Bcl-6 antibody (Abcam, USA) at 37°C for 2 h, followed by incubation with an HRP-conjugated goat anti-mouse IgG (Sigma, USA) or FITC-conjugated goat anti-mouse IgG (ThermoFisher, USA). Signal development was followed by observation under inverted microscope.

To analyze the CIQ249-induced differentiation of IgA-secreting cells in PPs, a combination of flow cytometry and IHC was employed. Detailed methodologies for intestinal PPs isolation, single-cell preparation, and flow cytometry were as follows: for collection and processing of PPs, fresh intestinal segments containing visible PPs were aseptically excised from euthanized mice, fat tissue and mesentery were carefully removed under a stereomicroscope, and individual PPs were gently separated using fine forceps and immediately placed in cold, sterile PBS. For preparation of single-cell suspension, isolated PPs were transferred onto a sterile 300-mesh stainless-steel cell strainer and gently pressed with the plunger of a syringe while rinsing with cold PBS to release lymphocytes. The resulting cell suspension was collected and centrifuged at 500 × g for 5 min at 4°C. The pellet was resuspended in 5 mL of digestion medium consisting of RPMI-1640 supplemented with 1 mg/mL collagenase IV (Sigma) and 0.1 mg/mL DNase I (Sigma), followed by incubation at 37°C for 30 min with gentle shaking. After digestion, the cell suspension was passed through a 400-mesh cell strainer to remove debris and undigested tissue, washed twice with cold PBS and centrifuged at 500 × g for 5 min. The final pellet was resuspended in FACS buffer (PBS containing 2% fetal bovine serum and 1 mM EDTA) and adjusted to a concentration of 2 × 10^6^ cells/mL. For surface marker staining, 100 μL of cell suspension (2 × 10^5^ cells) was aliquoted into FACS tubes and incubated with the following antibody cocktails for 30 min at 4 °C in the dark: T-cell panel (for Tfh identification): experimental group: APC-conjugated anti-mouse CXCR5 (3 μL) + PE-conjugated anti-mouse CD4 (3 μL) (BD pharmingen, USA); single-color controls: APC-CXCR5 alone, and PE-CD4 alone; unstained control: cells + PBS only. B-cell/plasma-cell panel (for IgA-secreting lineage analysis): experimental group: FITC-conjugated anti-mouse IgA (4 μL) + PerCP-Cy5.5-conjugated anti-mouse B220 (2 μL) + PE-Cy7-conjugated anti-mouse IgM (2 μL) (BD pharmingen, USA); single-color controls: FITC-IgA alone, PerCP-B220 alone, and PE-Cy7-IgM alone; Unstained control: cells + PBS only. After incubation, cells were washed twice with cold FACS buffer, centrifuged at 500 × g for 5 min, and finally resuspended in 500 μL of PBS for immediate acquisition. Subsequently, the percentage of CXCR5^+^CD4^+^ T follicular helper (Tfh) cells, B220^+^IgM^+^ B lymphocytes, B220^+^IgA^+^ B lymphocytes, and B220^−^IgA^+^ plasma cells were analyzed on a flow cytometer (BD Biosciences), and the gating strategy was as follows—live cell gate: based on forward scatter (FSC-A) vs. side scatter (SSC-A) to exclude debris and dead cells; single-cell gate: FSC-H vs. FSC-A to exclude doublets; T-cell subset: from single cells, CD4^+^ cells were gated, and subsequent analysis of CXCR5 expression identified CD4^+^CXCR5^+^ Tfh cells; B-cell/plasma-cell subset: from single cells, B220^+^IgM^+^ conventional B cells, B220^+^IgA^+^ IgA-committed B cells, and B220^−^IgA^+^ IgA-secreting plasma cells were identified based on co-expression of B220, IgM, and IgA. Additionally, IgA-secreting cells within the PPs were visualized by IHC, as described in the previous section, using an anti-mouse IgA antibody (Abcam, USA). All experiments were performed with three independent biological replicates.

### Transcriptome analysis of CIQ249-treated PIEC

PIEC were subjected to treatments as follows:(1) Control (sterile PBS) group; (2) CIQ249 alone (2 × 10^9^ CFU/mL for 3 h); (3) *S. typhimurium* alone (2 × 10^7^ CFU/mL for 3 h); (4) strain CIQ249 for 3 h, then infection with *S. typhimurium* for 3 h. Subsequently, cells from each group were collected and sent for transcriptomic analysis by Guangzhou Genedenovo Co., Ltd. (Guangzhou, China). All experiments were performed with three independent biological replicates per condition (control, strain CIQ249, *S. typhimurium*, and CIQ249+*S. typhimurium*).

### Statistical analysis

Data are presented as mean ± standard deviation (SD). Differences among groups were analyzed using one-way ANOVA followed by Tukey’s multiple-comparison test in GraphPad Prism V8.0. Significant differences are indicated with asterisks (*, P < 0.05; **, P < 0.01; ***, P < 0.001; ****, P < 0.0001). All experiments were performed in at least triplicate.

## Results

### Biological characteristics of *L. rhamnosus* CIQ249

**Figure 1:**
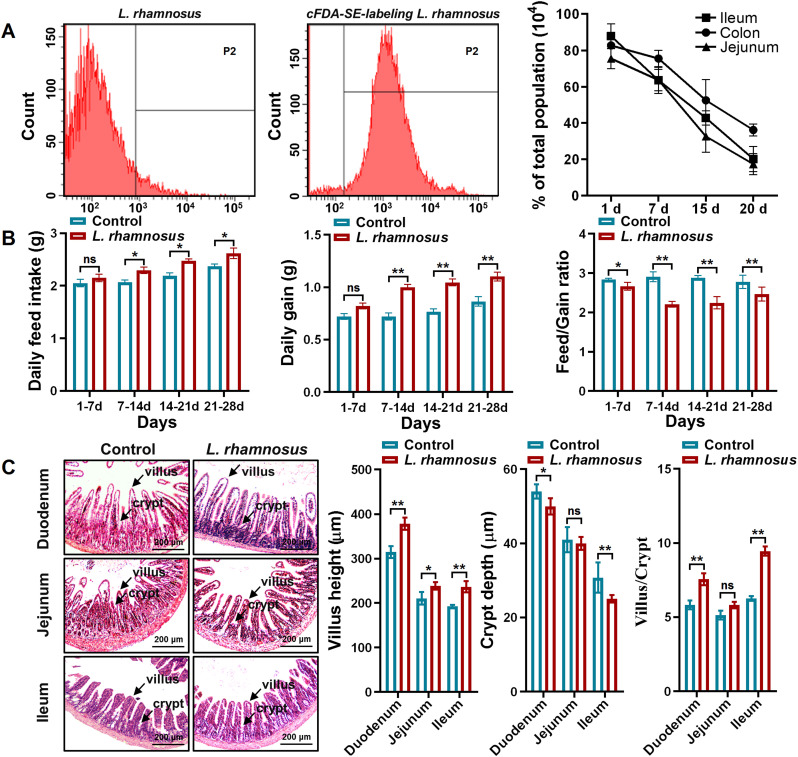
Biological characteristics of *L. rhamnosus* CIQ249. **(A)** Flow cytometry analysis showing the labeling efficiency of CFDA-SE and the intestinal colonization persistence of *L. rhamnosus* CIQ249 in mice. **(B)** Effect of *L. rhamnosus* CIQ249 supplementation on average daily feed intake, average daily gain, and feed-to-gain ratio in mice. **(C)** Representative H&E-stained sections of the intestine and quantification of villus height, crypt depth, and the villus-to-crypt (V/C) ratio in different intestinal segments of mice. Data are representative of three independent experiments and are presented as mean ± SD; *P < 0.05, **P < 0.01.

Flow cytometry confirmed approximately 99.3% positive labeling rate for cFDA-SE-labeled CIQ249 (**Figure 1A**). The strain demonstrated significant intestinal colonization, with detection rates of 75.03%, 87.82%, and 82.75% in the jejunum, ileum, and colon, respectively, on day 1 post-administration, and remained detectable (~20%) up to day 20 (**Figure 1A**). In mice, the strain CIQ249 markedly increased average daily feed intake and average daily gain while reducing the feed-to-gain ratio (**Figure 1B**). Furthermore, it promoted villus growth in the duodenum, jejunum, and ileum, resulting in shallower crypts and a significantly increased V/C ratio in the duodenum and ileum (**Figure 1C**).

### Antibacterial effect of *L. rhamnosus* CIQ249-free culture supernatant

**Figure 2:**
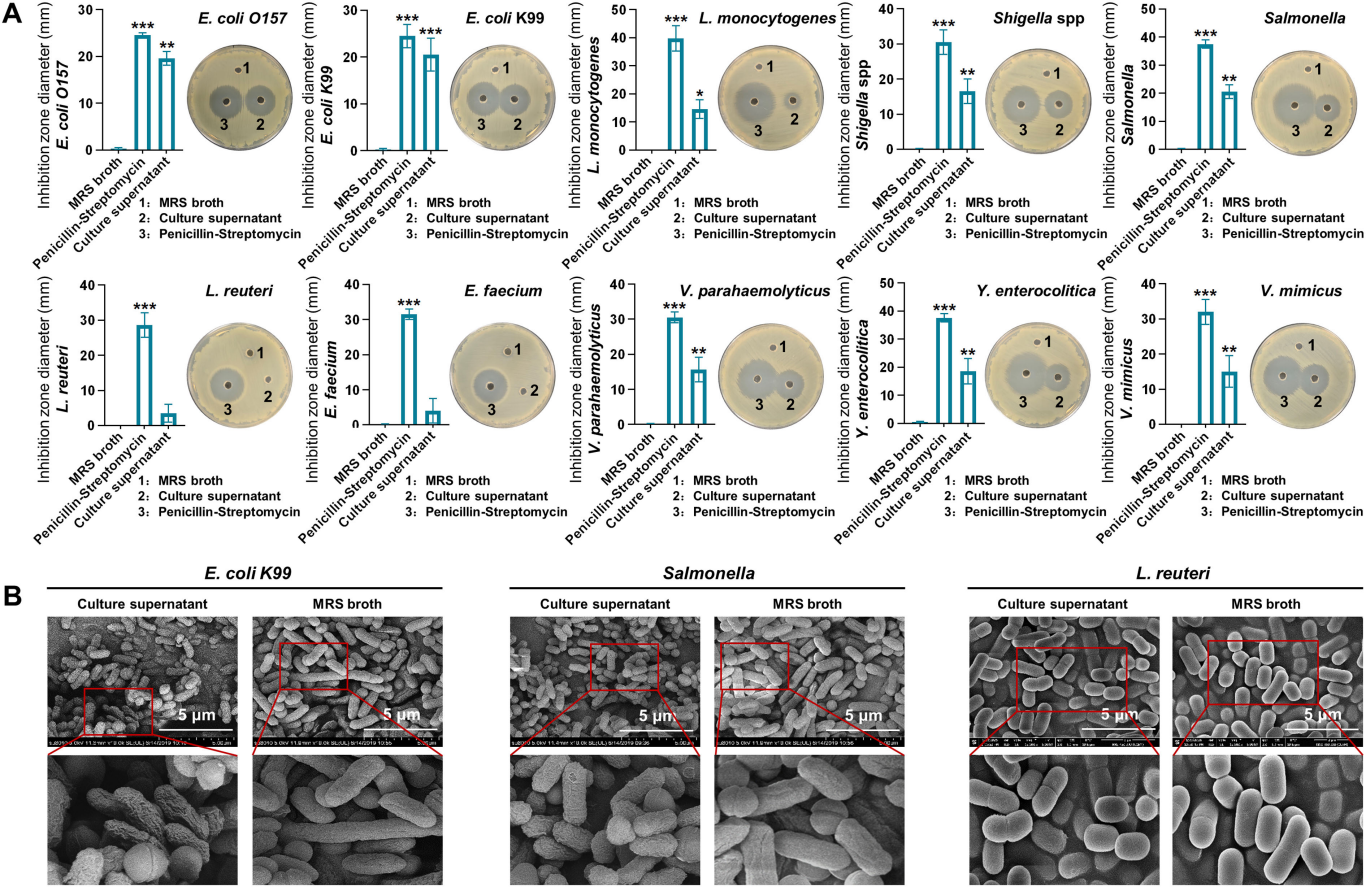
Antimicrobial activity of the CIQ249-free culture supernatant *in vitro*. **(A)** Inhibitory zones of *L. rhamnosus* CIQ249 culture supernatant (pH 7.0) against various pathogenic and probiotic bacteria. Penicillin/Streptomycin served as a positive control. **(B)** Scanning electron microscopy images showing morphological damage to *E coli* K99 and *S. typhimurium* after incubation with the *L. rhamnosus* CIQ249 supernatant, while the morphology of the probiotics L. reuteri remained intact, with no signs of shrinkage or damage. Data are representative of three independent experiments and are presented as mean ± SD; *P < 0.05, **P < 0.01, ***P < 0.001.

**Figure 3:**
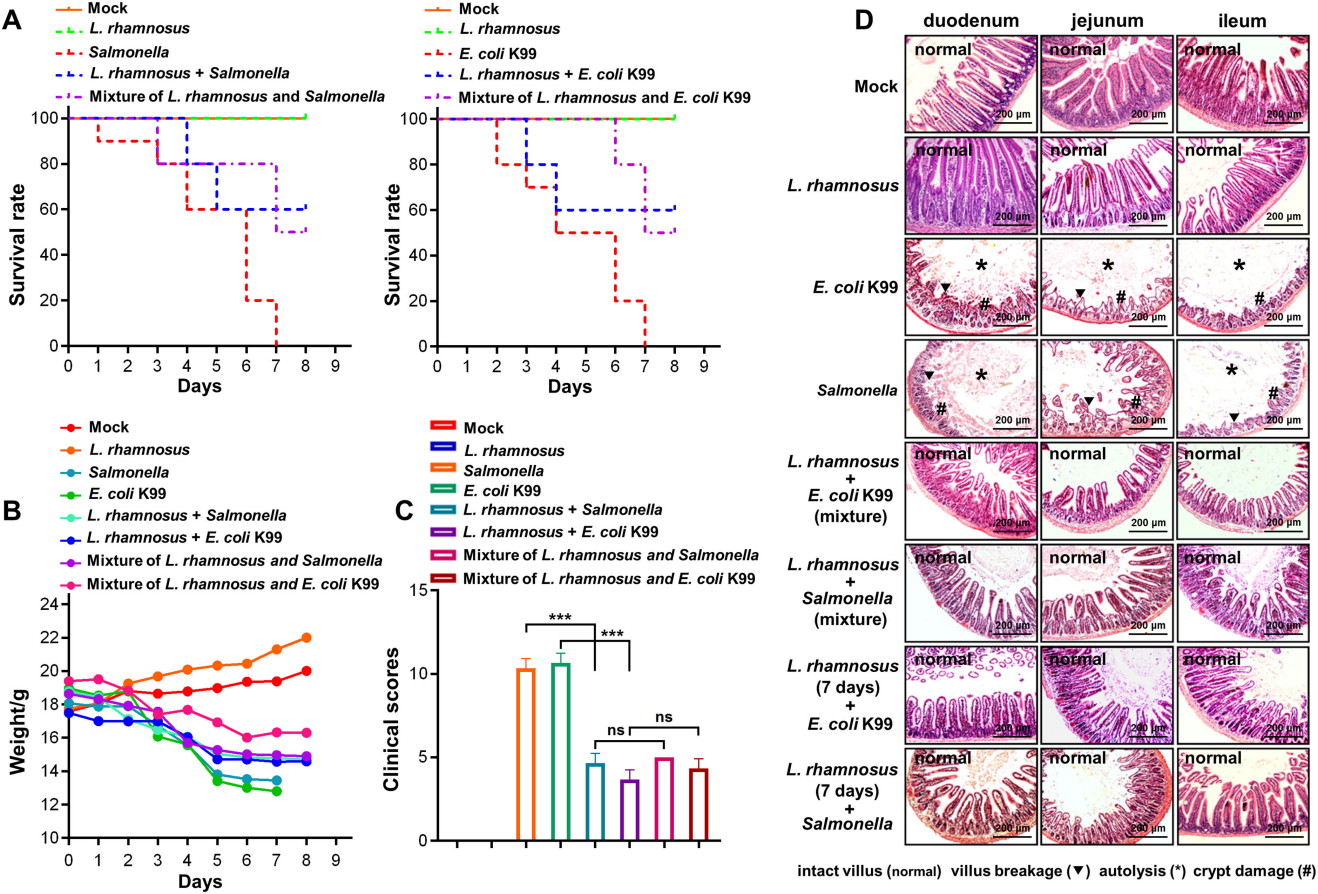
Protective efficacy of *L. rhamnosus* CIQ249 against pathogens infection in mice. **(A)** Survival curves of mice in different treatment groups following challenge with *S. typhimurium* or *E coli* K99. **(B)** Body weight changes of mice during the experimental period. **(C)** Clinical symptom scores of mice based on the scale defined in **Supplementary Table S1**. **(D)** Representative H&E-stained sections of mouse intestine obtained from each group. Data are representative of three independent experiments and are presented as mean ± SD; *P < 0.05, ***P < 0.001.

The filtered supernatant (pH 7.0) of the CIQ249 culture exhibited inhibitory activity against various pathogenic bacteria but had not effect on the probiotics *L. reuteri* and *E. faecium* (**Figure 2A**). Scanning electron microscopy (SEM) analysis revealed distinct shrinkage and damage to the cell walls of *E. coli* K99 and *S. typhimurium* following incubation with the CIQ249 supernatant (**Figure 2B**). In contrast, the morphology of the probiotics *L. reuteri* remained intact, with no signs of shrinkage or damage. These observations suggest that the damage to pathogens is not a nonspecific result of nutrient depletion but rather reflects the specific action of antagonistic substances present in the CIQ249 supernatant. In the mouse infection model, no mortality occurred in groups receiving CIQ249 alone or as a pre-treatment (**Figure 3A**). In contrast, mice infected with *S. typhimurium* or *E. coli* K99 alone began dying on days 1-2, with 100% mortality by day 7. Co-administration groups showed > 50% survival. Body weight loss was severe in pathogen-only groups but significantly attenuated in groups receiving the CIQ249 (**Figure 3B**). Clinical symptom scores were markedly lower in groups receiving the CIQ249 (either as pre-treatment or co-administration) compared to pathogen-only groups (**Figure 3C**). Histopathology confirmed severe villus breakage, autolysis, and crypt damage in *S. typhimurium* and E. coli K99-infected mice, while intestinal architecture remained relatively intact in CIQ249-treated groups (**Figure 3D**).

### Regulation of the expression of tight junction proteins ZO-1 and Claudin-1 by *L. rhamnosus* CIQ249

**Figure 4:**
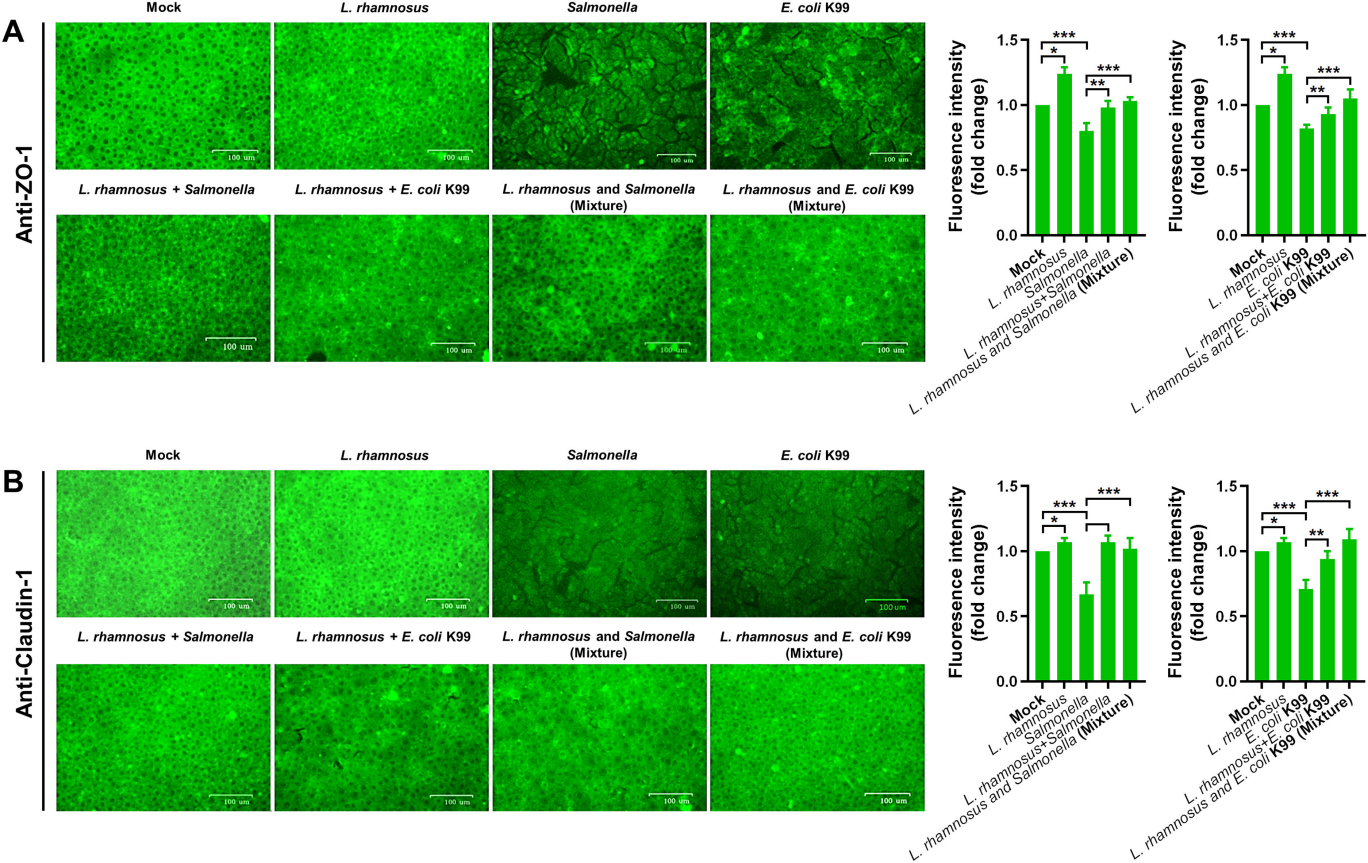
*L. rhamnosus* CIQ249 preserves tight junction integrity in porcine intestinal epithelial cells (PIEC). Indirect immunofluorescence staining for the tight junction proteins **(A)** ZO-1 and **(B)** Claudin-1 in PIEC under various treatment conditions. *P < 0.05, **P < 0.01, ***P < 0.001.

**Figure 5:**
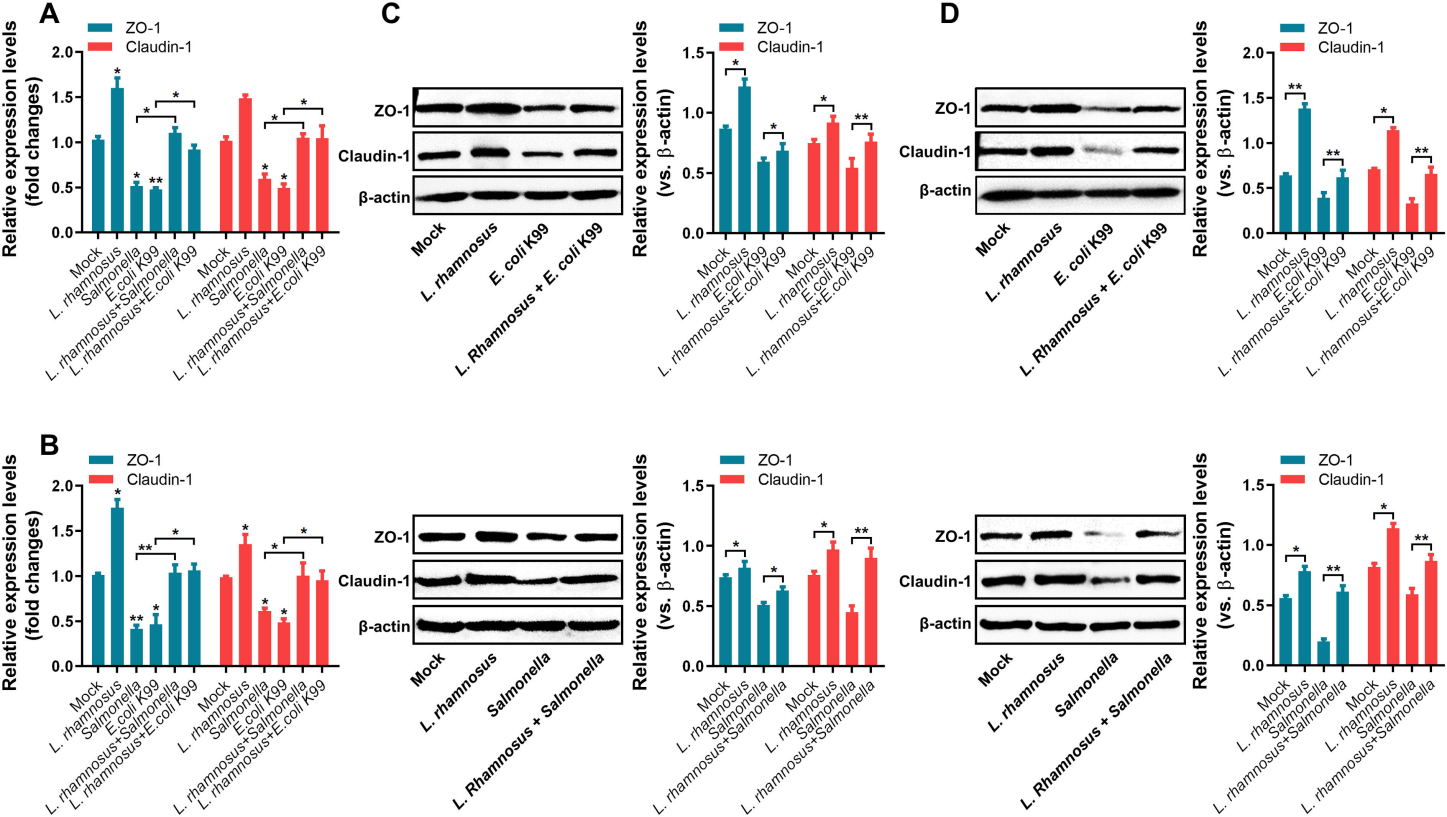
*L. rhamnosus* CIQ249 regulates the expression of tight junction proteins. mRNA expression levels of ZO-1 and Claudin-1 in **(A)** PIEC and **(B)** mouse intestinal tissues, as determined by RT-qPCR. Protein expression levels of ZO-1 and Claudin-1 in **(C)** PIEC and **(D)** mouse intestinal tissues, as analyzed by Western blot, using β-actin as the loading control. Data are representative of three independent experiments and are presented as mean ± SD; *P < 0.05, **P < 0.01.

Indirect immunofluorescence showed diffuse and discontinuous staining of ZO-1 (**Figure 4A**) and Claudin-1 (**Figure 4B**) with intercellular breaks in PIEC treated with *S. typhimurium* or *E. coli* K99 alone. In contrast, the CIQ249 treatment maintained or restored continuous, intact tight junction protein localization, even upon pathogenic challenge. At the gene level, the CIQ249 significantly upregulated ZO-1 and Claudin-1 mRNA expression in both PIEC (**Figure 5A**) and mouse intestinal cells (**Figure 5B**) compared to controls, whereas pathogens downregulated their expression. Pre-treatment with the CIQ249 prevented the pathogen-induced downregulation. Consistent with the transcriptional regulation, Western blot revealed corresponding changes at the protein level. Densitometric quantification of bands, normalized to β-actin, was performed across biological replicates. In PIEC (**Figure 5C**), treatment with CIQ249 significantly upregulated the expression of ZO-1 and Claudin-1. In contrast, challenge with *S. typhimurium* or *E. coli* K99 alone markedly reduced the levels of both proteins. Pre-incubation with CIQ249 prior to pathogen challenge effectively prevented this downregulation. A similar protective pattern was observed *in vivo* in mouse intestinal tissues (**Figure 5D**). Oral administration of CIQ249 alone for 5 days increased both proteins expression compared to the PBS-treated control. Infection with *S. typhimurium* or *E. coli* K99 alone led to a significant reduction. Importantly, pre-treatment with CIQ249 completely abolished the pathogen-induced decrease, restoring the expression of both tight junction proteins.

### Modulation of cellular immune response and IgA antibody secretion by *L. rhamnosus* CIQ249

**Figure 6:**
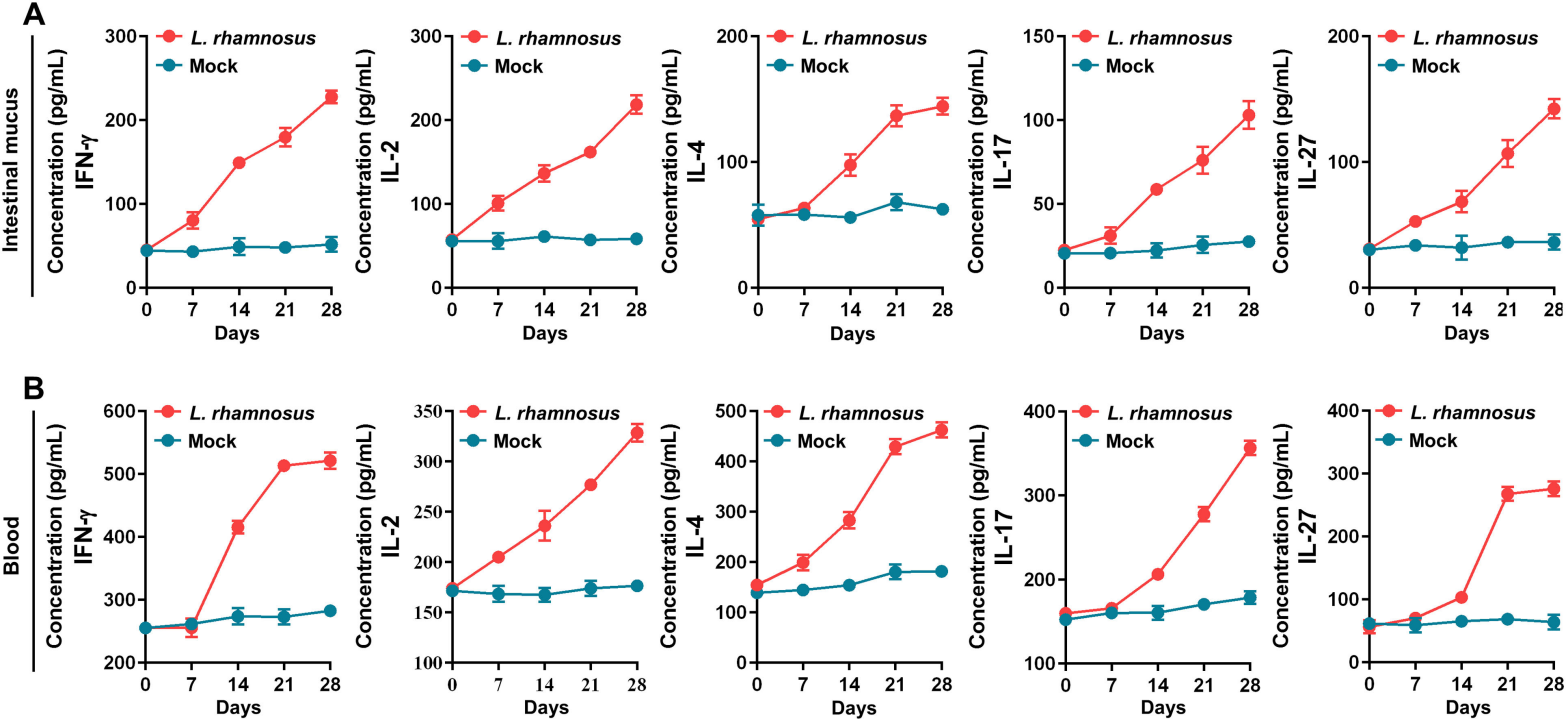
*L. rhamnosus* CIQ249 modulates systemic and mucosal cytokine production. Levels of IFN-γ, IL-2, IL-4, IL-17, and IL-27 in **(A)** the intestinal mucus and **(B)** serum of mice, measured by ELISA at different time points after oral administration of *L. rhamnosus* CIQ249. Data are representative of three independent experiments and are presented as mean ± SD.

**Figure 7:**
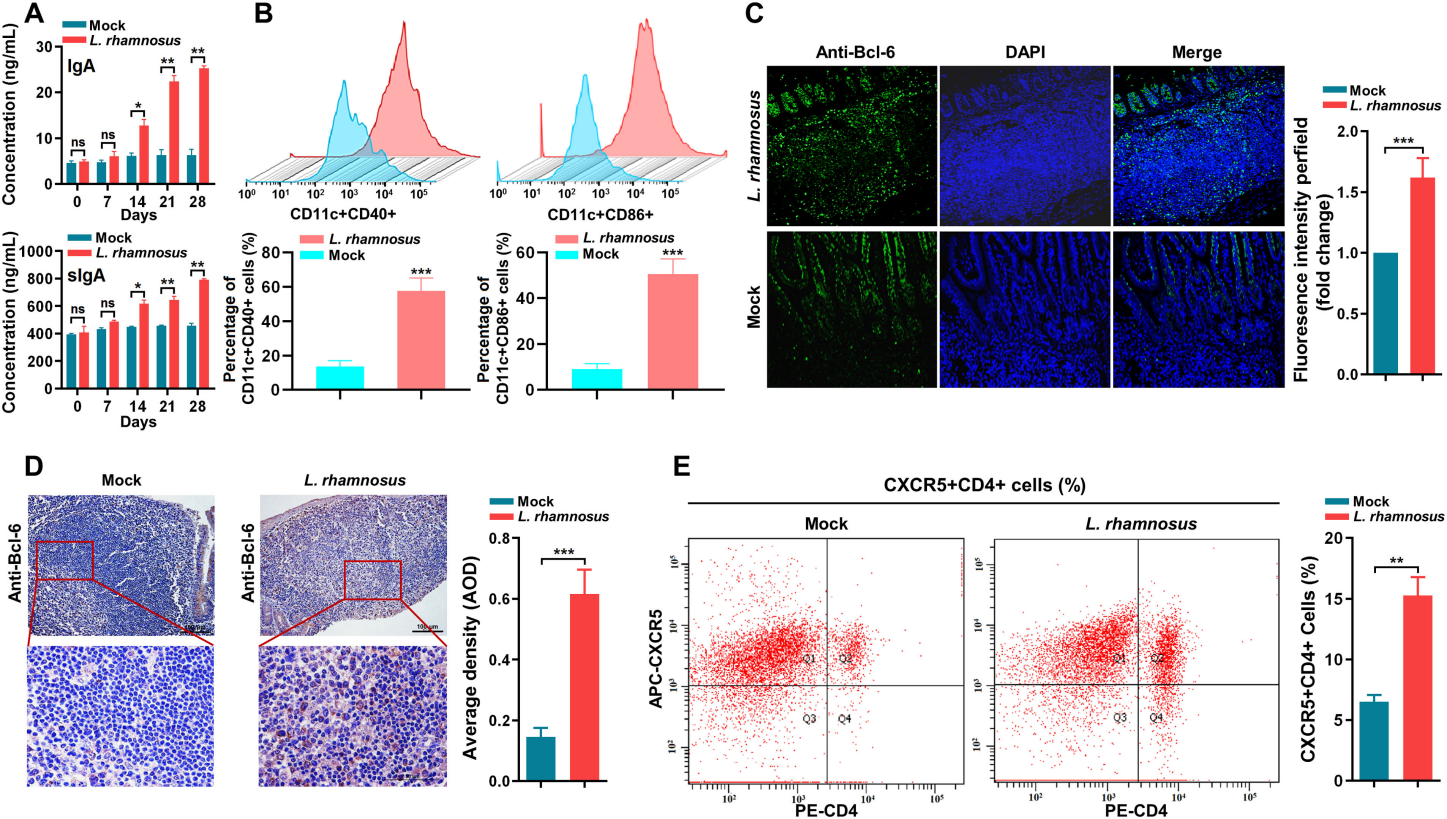
*L. rhamnosus* CIQ249 enhances mucosal humoral immunity and immune cell activation. **(A)** Serum IgA and intestinal secretory IgA (sIgA) levels in mice. **(B)** Flow cytometry analysis of activated dendritic cells (CD11c^+^CD40^+^ and CD11c^+^CD86^+^) in intestinal Peyer’s patches. **(C)** Fluorescence immunohistochemical and **(D)** immunohistochemical and detection of Bcl-6 expression in T cells within Peyer’s patches. **(E)** Flow cytometry analysis of T follicular helper (Tfh) cells (CXCR5^+^CD4^+^) in Peyer’s patches. Data are representative of three independent experiments and are presented as mean ± SD; *P < 0.05, **P < 0.01, ***P < 0.001.

**Figure 8:**
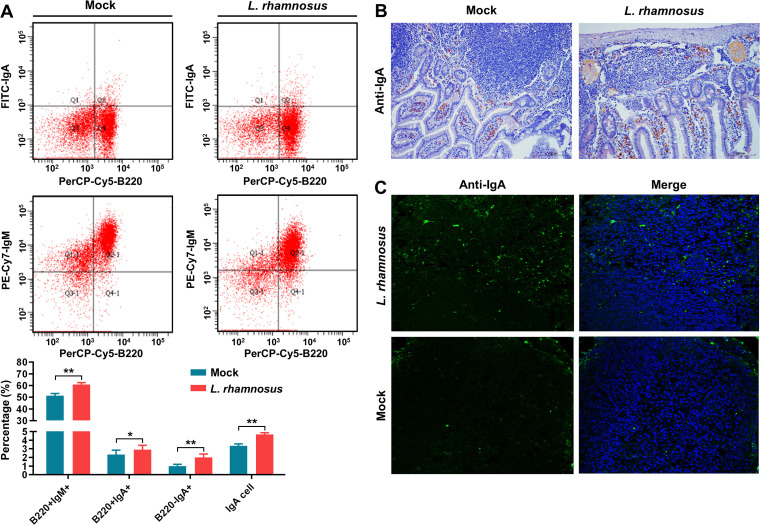
*L. rhamnosus* CIQ249 promotes B cell differentiation and IgA secretion. **(A)** Flow cytometry analysis of B cell populations (B220^+^IgM^+^, B220^+^IgA^+^, B220^-^IgA^+^) in Peyer’s patches. **(B)** Immunohistochemical and **(C)** fluorescence immunohistochemical staining showing IgA-secreting cells in the intestine. Data are representative of three independent experiments and are presented as mean ± SD; *P < 0.05, **P < 0.01.

Oral administration of the strain CIQ249 for 28 days gradually increased the levels of cytokines IFN-γ, IL-2, IL-4, IL-17, and IL-27 in both intestinal mucus (**Figure 6A**) and serum (**Figure 6B**). Serum IgA and intestinal sIgA levels were significantly elevated from day 14 onwards in the CIQ249 group compared to controls (**Figure 7A**). Flow cytometry revealed a significant increase in activated intestinal dendritic cells, evidenced by higher numbers of CD11c^+^CD40^+^ and CD11c^+^CD86^+^ cells (**Figure 7B**). Immunohistochemistry (**Figure 7C**) and fluorescence IHC (**Figures 7D**) showed increased Bcl-6 expression in T cells within the PPs of CIQ249-treated mice. Subsequently, flow cytometry confirmed a significant increase in CXCR5^+^CD4^+^ T follicular helper (Tfh) cells in PPs (**Figure 7E**). Furthermore, *L. rhamnosus* CIQ249 administration increased the percentages of B220^+^IgM^+^, B220^+^IgA^+^, and B220^−^IgA^+^ cells in PPs (**Figure 8A**), thereby promoting the differentiation of IgA-secreting cells, as corroborated by immunohistochemical analysis (**Figures 8B, C**).

### Transcriptome sequencing analysis of CIQ249-treated PIEC and qRT-PCR validation

**Figure 9:**
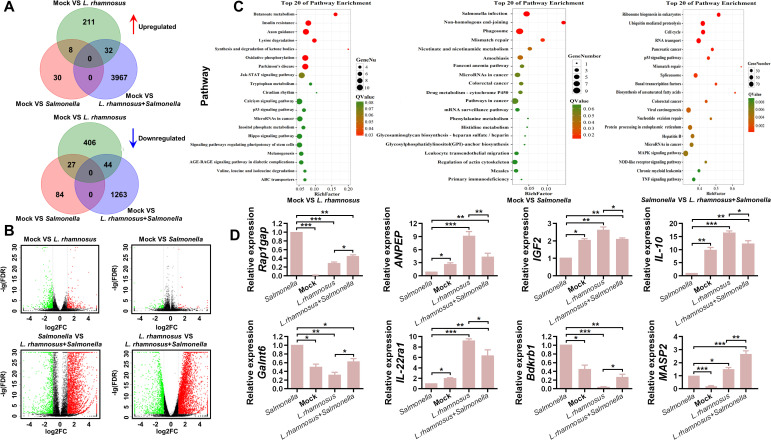
Transcriptomic analysis of *L. rhamnosus* CIQ249-treated PIEC. **(A)** Venn diagram illustrating the number of unique and shared differentially expressed genes (DEGs) among the comparison groups. **(B)** Volcano plots visualizing DEGs. Red dots represent upregulated genes, blue dots represent downregulated genes (cutoff: |log2FC| > 2, p < 0.05). **(C)** KEGG pathway enrichment analysis was conducted for the following comparisons: control vs. *L. rhamnosus*, control vs. *Salmonella*, and *Salmonella* vs. *L. rhamnosus* + *Salmonella*. **(D)** Validation of mRNA levels of eight selected DEGs (*Rap1gap*, *Anpep*, *Igf2*, *IL-10*, *Galnt6*, *IL-22ra1*, *Bdkrb1*, *Masp2*) from RNA-seq analysis by RT-qPCR. Data are representative of three independent experiments and are presented as mean ± SD; *P < 0.05, **P < 0.01 ***P < 0.001.

In this work, the relationships between samples were visualized using correlation heatmaps. As shown in **Supplementary Figure S1**, the R² values between samples within each group were all greater than 0.8, indicating good reproducibility of the samples and supporting the feasibility of subsequent analyses. RNA-seq analysis yielded high-quality data (clean reads coverage > 95.57% alignment rate > 63.39%). Differential gene expression analysis (cutoff: *P* < 0.05 and |log2FC| > 2) identified 211 upregulated and 406 downregulated unique genes in the *L. rhamnosus* group vs. control; 30 upregulated and 84 downregulated genes in the *Salmonella* group vs. control; and 3987 upregulated and 1263 downregulated genes in the *L. rhamnosus* + *Salmonella* group vs. the *Salmonella* group (**Figures 9A, B**). To gain deeper mechanistic insight, a more informative KEGG pathway enrichment analysis was conducted for the following comparisons: control vs. *L. rhamnosus*, control vs. *Salmonella*, and *Salmonella* vs. *L. rhamnosus* + *Salmonella* (**Figure 9C**), and these data indicated that the top enriched pathways were predominantly associated with broad metabolic and immune response processes. Additionally, to confirm the RNA-seq trends, eight genes—*Rap1gap*, *Anpep*, *Igf2*, *IL-10*, *Galnt6*, *IL-22ra1*, *Bdkrb1*, and *Masp2*—were selected for validation by RT-qPCR. These genes were among the most significantly differentially expressed in the RNA-seq dataset and were functionally implicated in key biological processes relevant to this study: immune regulation *(IL-10*, *IL-22ra1*), inflammation (*Bdkrb1*), complement activation (*Masp2*), growth signaling (*Igf2*), epithelial function/metabolism (*Anpep*, *Galnt6*), and cell signaling (*Rap1gap*). As shown in **Figure 9D**, the RT-qPCR results corroborated the sequencing data, supporting the reliability of the transcriptomic findings.

## Discussion

Intestinal pathogens pose a major threat to the livestock and poultry health. While antibiotics have been the primary therapeutic strategy, the rise of antimicrobial resistance underscores the critical need for effective alternatives (Ayaz et al., 2019; Krieger et al., 2020; Li et al., 2015). Probiotics, which beneficially modulate host health when consumed in adequate amounts, represent promising antibiotic substitutes. Given the established probiotic properties of *L. rhamnosus*, a systematic investigation into its mechanisms of action against enteric pathogens is essential for its optimized application.

A key prerequisite for probiotic efficacy is the ability to survive the gastrointestinal tract and colonize the intestine. *L. rhamnosus* CIQ249 demonstrated strong intestinal colonization persistence, consistent with its known acid and bile tolerance (Gu et al., 2022). This colonization was associated with improved growth performance in animals, as reflected by increased ADFI, ADG, and improved F/G ratio. Enhanced intestinal morphology, including increased villus height and V/C ratio, likely contributed to improved nutrient absorption and barrier function (Li et al., 2019; Wilson et al., 2018), aligning with reports on other probiotics like Bacillus subtilis (Ding et al., 2021).

The antimicrobial activity of probiotics often involves the production of metabolites. Our study confirmed that CIQ249-free culture supernatant inhibits a range of enteropathogens, consistent with previous reports on lactic acid bacteria (Hu et al., 2022). SEM visualization provided direct evidence of CIQ249 extracellular products-induced morphological damage to pathogenic bacteria. Importantly, these CIQ249 extracellular products did not inhibit the probiotic strains *E. faecium* and *L. reuteri*, suggesting a targeted antimicrobial effect. *In vivo*, the CIQ249 conferred significant protection against lethal challenges with *Salmonella* spp. and *E. coli* K99, improving survival, mitigating clinical symptoms, and preserving intestinal histoarchitecture.

The intestinal epithelial barrier, maintained by tight junction proteins like ZO-1 and Claudin-1, is crucial for preventing pathogen translocation (Thoo et al., 2019; Chelakkot et al., 2018; Haas et al., 2020; Garcia-Hernandez et al., 2017; Resta-Lenert and Barrett, 2003). Pathogens often disrupt these junctions. We found that *L. rhamnosus* CIQ249 not only promoted the expression of ZO-1 and Claudin-1 at both mRNA and protein levels but also counteracted the disruptive effects of pathogens on tight junction integrity *in vitro* and *in vivo*. This protective effect on the physical barrier may be facilitated by the adhesion of probiotics to epithelial cells, a known trait of *L. rhamnosus* (Poimenidou et al., 2023).

Beyond the physical barrier, *L. rhamnosus* CIQ249 robustly enhanced mucosalimmune responses. It elevated the levels of key cytokines (IFN-γ, IL-2, IL-4, IL-17, and IL-27) involved in orchestrating anti-infective immunity (Xiang et al., 2021; Yang et al., 2021; Woodward et al., 2010; Temerozo et al., 2023). Notably, it significantly boosted both systemic IgA and mucosal sIgA production. sIgA is a cornerstone of mucosal immunity, neutralizing pathogens and preventing their adherence (Chairatana and Nolan, 2017; Iijima et al., 2001; Pietrzak et al., 2020). Our mechanistic data revealed that *L. rhamnosus* CIQ249 activated intestinal DCs, promoted Tfh cell differentiation (via increased Bcl-6 expression and CXCR5^+^CD4^+^ cell numbers), and subsequently drove B cell proliferation and differentiation into IgA-secreting plasma cells within Peyer’s patches. This coordinated action highlights the immunomodulatory prowess of *L. rhamnosus* CIQ249 in enhancing the humoral arm of mucosal immunity.

Transcriptome analysis provided a systems-level view of the host response. The dramatic shift in gene expression profile when *L. rhamnosus* was administered prior to *Salmonella* infection, compared to infection alone, indicates a profound modulatory effect. The upregulation of genes like *Masp2* (complement activation) (Møller-Kristensen et al., 2007), *Anpep* (potential immunomodulation) (Dar et al., 2022), *IL-10* (anti-inflammatory) (Li et al., 2022), and *Igf2* (cell growth) (Luo et al., 2023) suggests enhanced antibacterial defense, anti-inflammatory activity, and tissue repair. Conversely, the downregulation of genes like *Bdkrb1* (inflammation) (El Oumeiri et al., 2023), *Galnt6* (associated with invasiveness) (Mao et al., 2019), and *Rap1gap* (linked to negative growth regulation) (Xu et al., 2020) points to a suppression of detrimental inflammatory and pathological pathways.

**Figure 10:**
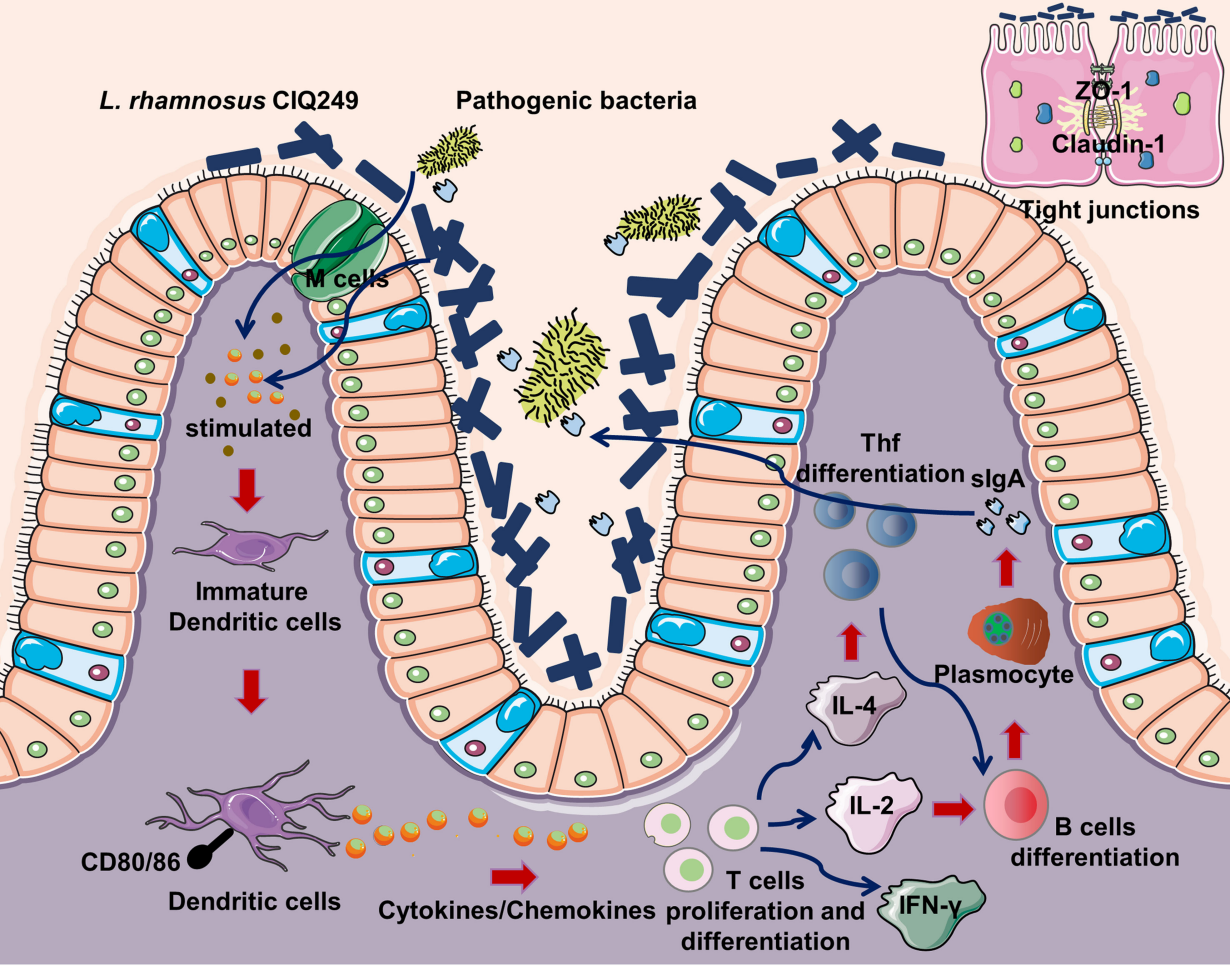
Schematic diagram of the mechanism by which *L. rhamnosus* CIQ249 improves intestinal mucosal immunity and epithelial barrier function to protect against enteropathogenic bacteria infections.

In conclusion, our findings demonstrate that *L.* rhamnosus strain CIQ249 enhances mucosal defense against enteropathogenic bacteria infection by a dual mechanism—fortifying the epithelial barrier and orchestrating a mucosal immune axis involving dendritic cell (DC) activation, subsequent T follicular helper (Tfh) cell differentiation, and the promotion of IgA^+^ B cell responses (DC–Tfh–IgA axis) (as summarized in **Figure 10**), and modulating the global intestinal transcriptome towards an anti-infective and homeostatic state. These comprehensive insights solidify the potential of *L. rhamnosus* CIQ249 as a promising microecological agent candidate for combating intestinal infections. Furthermore, we emphasize that while *L. rhamnosus* is a well-known probiotic, this study provides a comprehensive, multi-level mechanistic dissection of the strain CIQ249, integrating *in vivo* colonization, growth promotion, direct pathogen antagonism, detailed analysis of epithelial barrier protection (with quantification), systematic elucidation of its immunomodulatory pathway (from DC activation to IgA secretion), and global transcriptomic profiling. This systems-level understanding of how a single strain concurrently enhances both physical and immune barriers is a significant advancement.

## Data Availability

The datasets presented in this study can be found in online repositories. The names of the repository/repositories and accession number(s) can be found below: https://figshare.com/, 30899597.
