# Supplementary material for: Lactobacillus rhamnosus confers protection against enteropathogenic bacteria by enhancing mucosal immunity and epithelial barrier function
Source: Front Cell Infect Microbiol. 2026 Feb 23;16:1769889. doi: 10.3389/fcimb.2026.1769889 (PMC12968290; doi:10.3389/fcimb.2026.1769889)
Supplement: Supplementary file 1 [file Table1.docx]

**Tables**

**Table S1.** Clinical symptom scoring scale for mice.

| **Score** | **Body weight change** | **Activity & Posture** | **Stool Consistency** | **Coat condition** | **Appetite** |
| --- | --- | --- | --- | --- | --- |
| 0  (Normal) | Gain or stable | Alert, active, normal posture | Normal, formed pellets | Smooth, glossy | Normal |
| 1  (Mild) | Loss: 1-5% | Slightly subdued, reduced activity | Soft, pasty (mild diarrhea) | Slightly ruffled, dull | Slightly reduced |
| 2  (Moderate) | Loss: 6-10% | Lethargic, hunched posture | Loose, unformed (moderate diarrhea) | Clearly ruffled, piloerection | Markedly reduced (anorexic) |
| 3  (Severe) | Loss: >10% | Prostrate, immobile, or shows tremors/convulsions | Watery diarrhea | Severely ruffled, soiled | Absent (complete anorexia) |

**Table S2.** Primers used in this study.

| Gene | Primer Sequences (5’-3’) | Accession number |
| --- | --- | --- |
| *Rap1gap* | F: TTCCCTCCCACCCTGCTTCAC | NM_001100713.1 |
|  | R: TCGTTGTTCATCCATCCTGCTTCC |  |
| *Anpep* | F: TCATAGCTCTGTCGGTGGTCTACG | NM_031012.1 |
|  | R: TGGTAGTTGAGGTGGTGGCTGAG |  |
| *Igf2* | F: AAGACGGGAGAAGAGAAGGGAGTG | U71085.1 |
|  | R: GGAAGGGAAGATAGGGCAGGAGAG |  |
| *IL-10* | F: TCCCTGGGTGAGAAGCTGAAG | NM_010548.2 |
|  | R: CACCTGCTCCACTGCCTTGC |  |
| *Galnt6* | F: GAAGACAAGACAGCGGTGGTGAG | NM_001172063.2 |
|  | R: GCATCTCCCAGCCAAAGGTCAG |  |
| *IL-22ra1* | F: CTCGTCGGCTTGCTCTGTTATCTG | BC140973.1 |
|  | R: CGTGTTCTTGGATGAAGCGTAGGG |  |
| *Bdkrb1* | F: AGAAGACTGGGACCTGCTGTATCG | NM_030851.1 |
|  | R: GTTGCTGCCACCACCACTGTC |  |
| *Masp2* | F: GCAGCGGAGGATGTGGATGAATG | NM_172043.1 |
|  | R: ACTCGGCAGGAGCAGTAGTAGC |  |
| *Zo-1* | F: CCATTCAGGTCGCTCGCATGAC  R: CATTGCTGGGCTGCTGTGGAG | AF169196.1 |
| *Claudin-1* | F: TGGCTTCTCTGGGATGGATCGG  R: CCTGAGCGGTCACGATGTTGTC | NM_021101.5 |
| *β-actin* | F: GCCAACCGTGAGAAGATGAC  R: AGGCATACAGGGACAGCACA | HQ154074.1 |

**Figures**

**Figure S1**


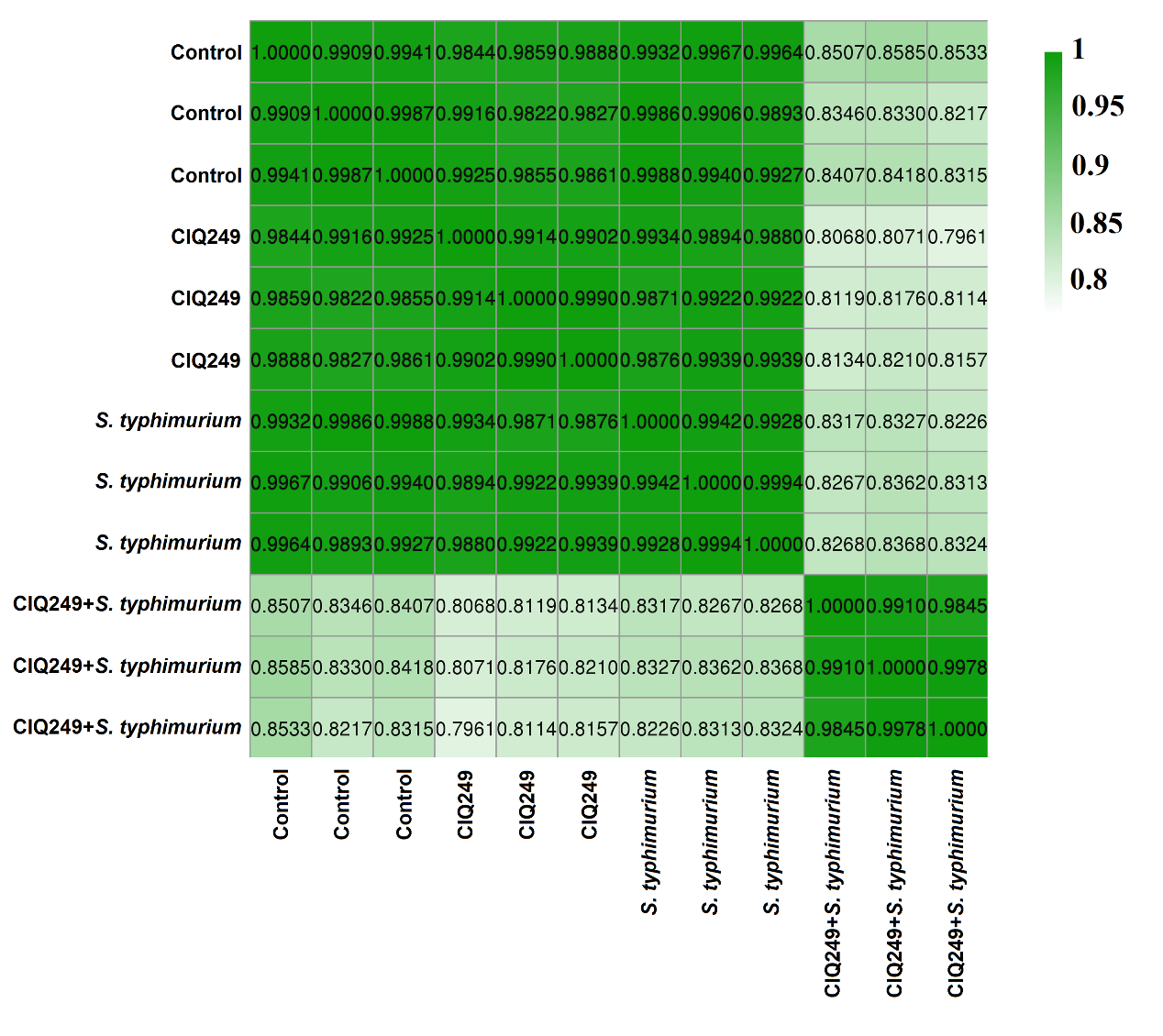


Figure S1 The relationships between samples visualized using correlation heatmaps
